# Supplementary material for: The interactome of intact mitochondria by cross-linking mass spectrometry provides evidence for coexisting respiratory supercomplexes
Source: Mol Cell Proteomics. 2017 Dec 8;17(2):216–32. doi: 10.1074/mcp.RA117.000470 (PMC5795388; doi:10.1074/mcp.RA117.000470)
Supplement: Supplemental Data [file supp_RA117.000470_133922_0_supp_23978_fzffwf.zip › spectra_annotation/mito_spectra_annotation/172-1-2-1-22-1.pdf]

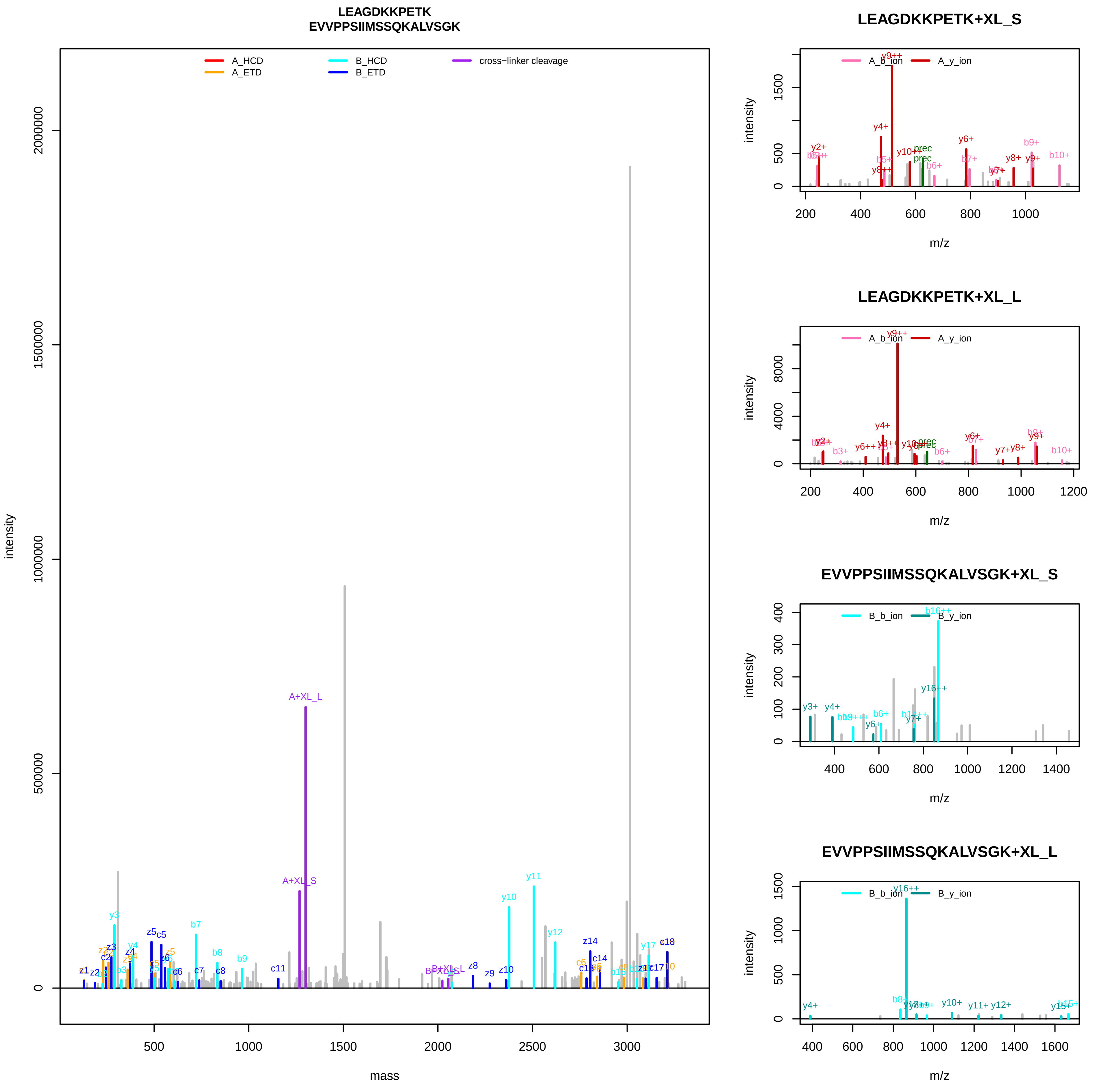

LEAGDKKPETK
EVVPPSIIMSSQKALVSGK
A_HCD
A_ETD
B_HCD
B_ETD
cross−linker cleavage
A+XL_L
A+XL_S
y3
y4
z3
z4
z5
c5
b7
b8
b9
c6
c7
c8
c11
y10
y11
y12
z8
z9
z10
z14
c14
y17
c18
2000000
1500000
1000000
500000
0
500
1000
1500
2000
2500
3000
intensity
mass
LEAGDKKPETK+XL_S
LEAGDKKPETK+XL_L
EVVPPSIIMSSQKALVSGK+XL_S
EVVPPSIIMSSQKALVSGK+XL_L
A_b_ion
A_y_ion
B_b_ion
B_y_ion
y9++
y4+
y2+
y6+
y8+
y9+
b9+
b10+
b6+
b7+
prec
y16++
y3+
b6+
y10+
y11+
y12+
y15+
intensity
m/z
